# Role of ST6GAL1 in Thyroid Cancers: Insights from Tissue Analysis and Genomic Datasets

**DOI:** 10.3390/ijms242216334

**Published:** 2023-11-15

**Authors:** Ivana Gunjača, Benjamin Benzon, Nikolina Pleić, Mirjana Babić Leko, Valdi Pešutić Pisac, Ana Barić, Dean Kaličanin, Ante Punda, Ozren Polašek, Katarina Vukojević, Tatijana Zemunik

**Affiliations:** 1 Department of Medical Biology, School of Medicine, University of Split, 21000 Split, Croatia; npleic@mefst.hr (N.P.); mbabic@mefst.hr (M.B.L.); dkalican@mefst.hr (D.K.); 2 Department of Anatomy, Histology and Embryology, School of Medicine, University of Split, 21000 Split, Croatia; benjamin.benzon@mefst.hr (B.B.); katarina.vukojevic@mefst.hr (K.V.); 3 Clinical Department of Pathology, Forensic Medicine and Cytology, University Hospital of Split, 21000 Split, Croatia; valdi.pesutic-pisac@mefst.hr; 4 Department of Nuclear Medicine, University Hospital of Split, 21000 Split, Croatia; ana.baaric@gmail.com (A.B.); ante.punda@mefst.hr (A.P.); 5 Department of Public Health, School of Medicine, University of Split, 21000 Split, Croatia; ozren.polasek@mefst.hr

**Keywords:** ST6GAL1, papillary thyroid cancer, follicular thyroid cancer, follicular variant of papillary thyroid carcinoma, microcarcinoma

## Abstract

Thyroid cancer is the predominant endocrine-related malignancy. ST6 β-galactoside α2,6-sialyltransferase 1 (ST6GAL1) has been studied in various types of cancers; however, the expression and function of ST6GAL1 in thyroid cancer has not been investigated so far. Previously, we conducted two genome-wide association studies and have identified the association of the *ST6GAL1* gene with plasma thyroglobulin (Tg) levels. Since Tg levels are altered in thyroid pathologies, in the current study, we wanted to evaluate the expression of ST6GAL1 in thyroid cancer tissues. We performed an immunohistochemical analysis using human thyroid tissue from 89 patients and analyzed ST6GAL1 protein expression in papillary thyroid cancer (including follicular variant and microcarcinoma) and follicular thyroid cancer in comparison to normal thyroid tissue. Additionally, *ST6GAL1* mRNA levels from The Cancer Genome Atlas (TCGA, n = 572) and the Genotype-Tissue Expression (GTEx) project (n = 279) were examined. The immunohistochemical analysis revealed higher ST6GAL1 protein expression in all thyroid tumors compared to normal thyroid tissue. TCGA data revealed increased *ST6GAL1* mRNA levels in both primary and metastatic tumors versus controls. Notably, the follicular variant of papillary thyroid cancer exhibited significantly higher *ST6GAL1* mRNA levels than classic papillary thyroid cancer. High *ST6GAL1* mRNA levels significantly correlated with lymph node metastasis status, clinical stage, and reduced survival rate. ST6GAL1 emerges as a potential cancer-associated glycosyltransferase in thyroid malignancies, offering valuable insights into its diagnostic and prognostic significance.

## 1. Introduction

The incidence of thyroid cancer has increased dramatically in recent decades [1]. It is the most common type of endocrine-related malignancy whose prevalence continues to rise; it is the fifth most common cancer in women. Tumor-associated changes can affect both follicular thyroid cells and neuroendocrine cells. Thyroid cancers that arise from follicular thyroid cells include well-differentiated thyroid cancer (papillary thyroid cancer, follicular thyroid cancer, and Hürthle cell thyroid cancer), poorly differentiated thyroid cancer, and anaplastic (undifferentiated) thyroid cancer. 

Well-differentiated thyroid carcinomas, predominantly comprising papillary thyroid cancer (PTC) and follicular thyroid cancer (FTC), represent the most prevalent forms of endocrine malignancy, characterized by their resemblance to normal thyroid tissue and a generally favorable prognosis. PTC, accounting for approximately 85% of cases, is characterized by unique papillary structures and often presents with painless nodules and potential cervical lymphadenopathy, with higher prevalence in younger females and a strong association with radiation exposure and certain genetic mutations. FTC, less common than PTC, is recognized for its hematogenous spread and potential vascular invasion. The mainstay of treatment for both cancers includes surgical resection, with adjuvant radioactive iodine therapy and thyroid hormone replacement as standard postoperative care. Despite their generally good outcome, a subset of well-differentiated thyroid carcinomas can exhibit aggressive behavior and may require more intensive therapy and follow up. Given the typically slow progression of these cancers, it remains crucial to maintain rigorous monitoring through imaging and serum biomarker assessments to promptly identify any signs of recurrence, thereby preserving the high survival rates associated with these conditions [1,2,3,4]. 

Genetic alterations, such as mutations in the *BRAF* and *RAS* genes or rearrangements of the *RET/PTC* and *PAX8/PPARγ*, are often implicated in their pathogenesis. Most observed mutations in thyroid cancers were in the mitogen-activated protein kinase (MAPK) pathway [5,6]. The most common mutations are in *BRAF* (60%) and *RAS* (15%) genes, followed by chromosomal translocations (12%; most commonly *RET-PTC* and *PAX8-PPARγ*) [1,2,3]. In addition to these somatic mutations, there are germline variants that can also contribute to the increased risk of thyroid cancer. In fact, it was shown that thyroid cancer has one of the strongest genetic components [7]. Genome-wide association studies (GWAS) commonly used to unravel the genetic basis of polygenic disorders were used in research on thyroid-cancer-specific germline variants [8,9,10,11,12,13,14,15,16,17,18]. Our recent GWAS identified that the gene encoding for ST6 beta-galactoside alpha-2,6-sialyltransferase 1 (ST6GAL1) was associated with plasma thyroglobulin (Tg) levels [19] and the result was confirmed in the replication study of our research group after including over a thousand new individuals, emphasizing that *ST6GAL1* significantly contributes to the variation of Tg plasma levels [20]. 

One of the main glycosylation processes altered in cancers is abnormal sialylation mediated with sialyltransferase enzymes [21]. Sialyltransferase enzymes catalyze the transfer of sialic acid via different glycosidic linkages (α2–3, α2–6, or α2–8) to terminal positions of glycoprotein and glycolipid carbohydrates [22]. Although there are 20 different human sialyltransferases, ST6GAL1 is amongst the most studied sialyltransferases in cancer research [21]. Levels of ST6GAL1 were upregulated in numerous types of cancers, including prostate [23], pancreatic [24], ovarian [25,26], colon [27], cervical [28], gastric [29], breast cancer [30], and glioma [31]. On the contrary, ST6GAL1 downregulation was observed in bladder cancer [32], but the expression of ST6GAL1 has not yet been analyzed in different types of thyroid cancer. Since Tg levels are altered in thyroid pathologies, in the current study, we wanted to analyze the ST6GAL1 protein expression in different types of well-differentiated thyroid cancers. The aim of our study was to observe the ST6GAL1 protein expression in papillary thyroid cancer (including follicular variant and microcarcinoma) and in follicular thyroid cancer, as well as to analyze the *ST6GAL1* mRNA levels in a larger thyroid cancer cohort retrieved from The Cancer Genome Atlas (TCGA) database. We additionally aimed to investigate the prognostic value of *ST6GAL1* mRNA levels as a potential prognostic marker for patient survival.

## 2. Results

We included thyroid tumor samples collected from 75 patients (17 men and 58 women; median age, 57; range, 40–66). We had tumor samples from 20 patients with follicular carcinoma (FTC), 20 patients with papillary carcinoma (PTC), 17 patients with a follicular variant of papillary carcinoma (FVPTC), and 18 patients with microcarcinoma. The control group was composed of 14 healthy thyroid tissue samples from patients (five men and nine women; median age, 48.5; range, 45–58) who had non-oncological thyroid surgery and no autoimmune thyroid disorders. The clinicopathological characteristics of patients are summarized in Table 1.

**Figure 1:**
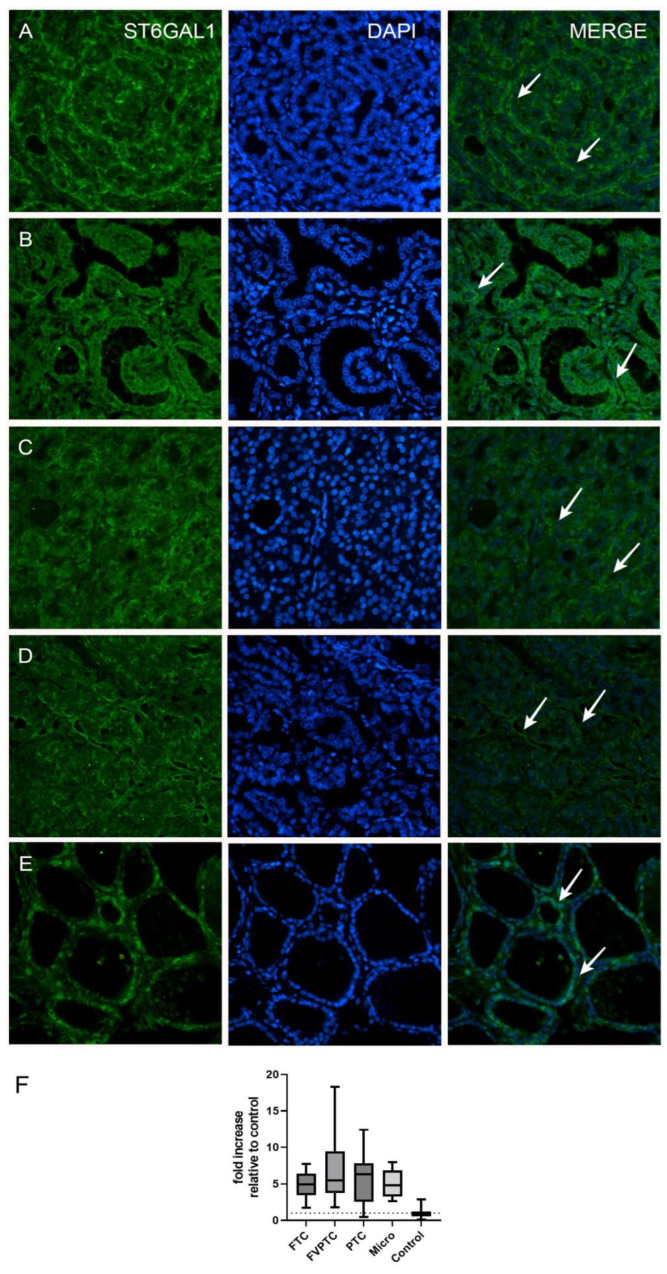
Immunofluorescent staining in (**A**) follicular variant of papillary thyroid cancer (FVPTC); (**B**) papillary thyroid cancer (PTC); (**C**) follicular thyroid cancer (FTC); (**D**) microcarcinoma; (**E**) normal thyroid tissue (control); all are with ST6GAL1 (arrows) and their co-expression with DAPI nuclear staining. All images were taken with a magnification of 40×. (**F**) Fold increases of ST6GAL1 expression relative to the control (healthy tissue) group in different types of thyroid cancer and healthy thyroid tissue (the dotted line represents a fold increase of 1, i.e., no increase relative to the control group).

**Figure 2:**
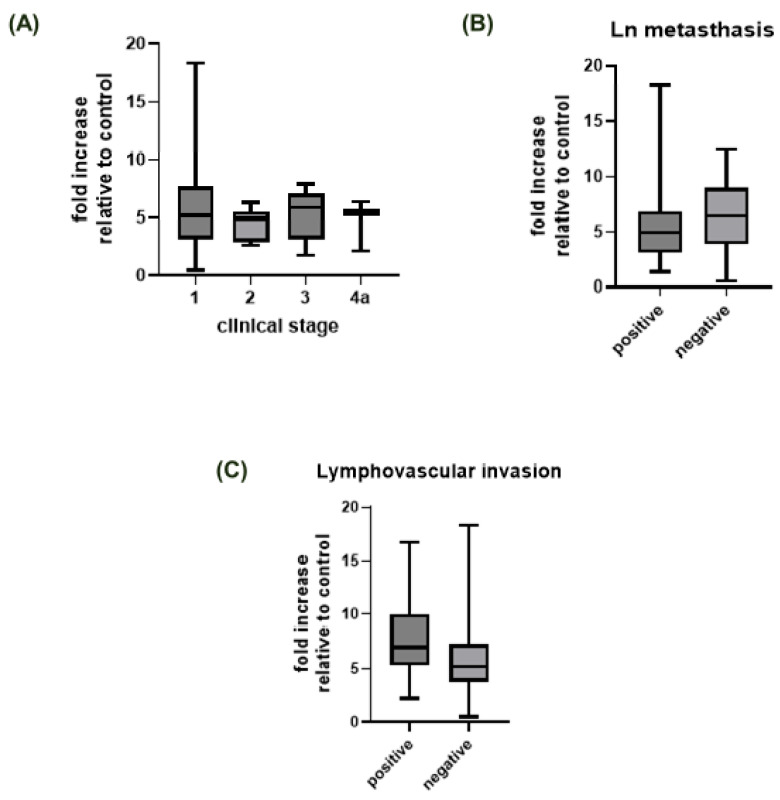
Fold increases in ST6GAL1 protein expression relative to the control (healthy tissue) group stratified by (**A**) clinical stage, (**B**) lymph node metastasis, and (**C**) invasion of blood or lymph vessels. The dotted line represents a fold increase of 1, i.e., no increase relative to the control group.

The analysis of ST6GAL1 protein expression in human thyroid tumors revealed that expression in thyroid tumors is higher in comparison to normal thyroid tissue (Table 1, Figure 1). On a more detailed basis, when compared to normal thyroid tissue, FVPTC had the highest ST6GAL1 protein expression (*p* = 0.0002), followed by classic PTC, FTC, and microcarcinoma (*p* < 0.0001) (Table 1, Figure 1). Stratification of patients by clinical stage, lymphovascular invasion, and lymph node metastasis status yielded no significant differences in ST6GAL1 protein expression (Table 1, Figure 2). 

### ST6GAL1 mRNA Levels in Thyroid Cancers

We analyzed the microarray data of the TCGA database to investigate the role of *ST6GAL1* mRNA levels in thyroid cancer progression. Out of the total TCGA sample (n = 572), we first excluded the Solid tissue normal samples (n = 59), as well as Thyroid Papillary Carcinoma—Tall Cell samples (n = 36) and unspecified thyroid carcinomas (n = 9). The resulting TCGA sample in our analysis consisted of 468 participants who were directly comparable to our samples (Table 2). We found that primary and metastatic tumors in the TCGA cohort had significantly higher levels of *ST6GAL1* mRNA compared to healthy controls (*p* = 0.008). Stratified by histological type, the FVPTC had significantly higher *ST6GAL1* mRNA levels compared to the classic PTC (*p* = 5.50 × 10^−6^). Also, there was a significant association of *ST6GAL1* mRNA levels with lymph node metastasis status (*p* = 1.57 × 10^−4^) and clinical stage (*p* = 0.036) (Table 2).

**Figure 3:**
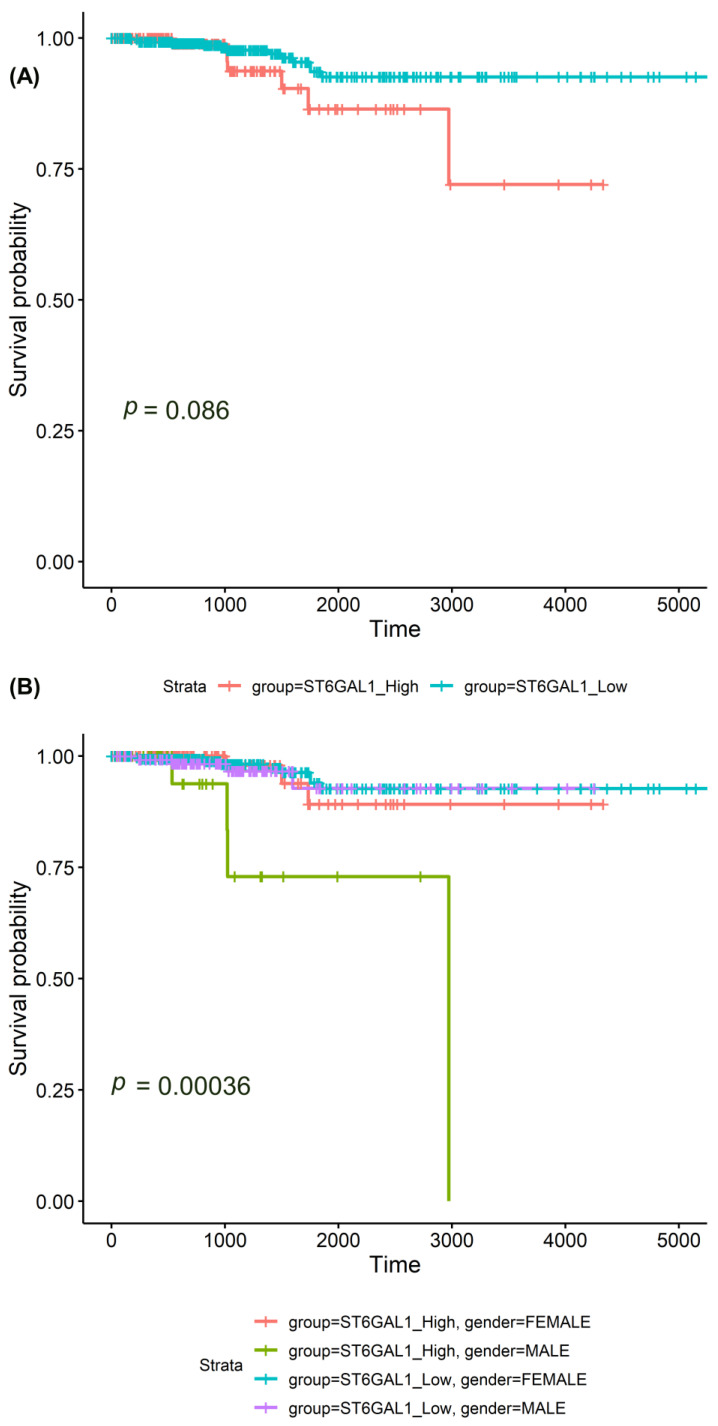
Survival analysis of the *ST6GAL1* mRNA levels in thyroid carcinomas (TCGA cohort). Kaplan–Meier analysis and log-rank tests show the following: (**A**) no significant difference between the survival rates of participants with high *ST6GAL1* mRNA levels (upper 25%) and low *ST6GAL1* mRNA levels; (**B**) a significant correlation of high *ST6GAL1* mRNA levels (upper 25%) with shorter overall survival in the subgroup of men (*p* = 0.0004).

In the survival analysis, we utilized the complete TCGA cohort, which has the data on the survival rates of the participants. We could not show a significant difference between the survival rates of participants with high *ST6GAL1* mRNA levels (upper 25%) and low *ST6GAL1* mRNA levels (Figure 3A). However, a stratified Kaplan–Meier analysis and a log-rank test showed a significant correlation of high *ST6GAL1* mRNA levels (upper 25%) with shorter overall survival in the subgroup of men (*p* = 0.0004, Figure 3B).

## 3. Discussion

Alternations in the glycosylation process are nowadays considered a new hallmark of cancer [33]. Sialylation mediated by sialyltransferase enzymes is one of the main glycosylation processes altered in cancer [21] and one principal sialyltransferase overexpressed in a plethora of malignant diseases is ST6GAL1 [34]. In this study, we analyzed the ST6GAL1 protein expression in different types of well-differentiated thyroid cancers and showed ST6GAL1 protein overexpression in all thyroid tumors compared to normal thyroid tissue. TCGA data also showed significantly higher ST6GAL1 mRNA levels in primary and metastatic tumors compared to healthy controls. Comparing tumors by histological type, the FVPTC had significantly higher ST6GAL1 protein and mRNA levels compared to the classic PTC variant. Many studies have associated high levels of ST6GAL1 expression with clinicopathological parameters such as advanced tumor grade, lymphovascular invasion, metastatic progression, and overall survival in different types of cancer [23,26,35]. The mRNA data from the TCGA database analyzed in our study also support the associations between ST6GAL1 mRNA expression and metastatic tumors, lymph node metastasis status, clinical stage, and shorter overall survival in thyroid cancers.

Studies on the involvement of sialyltransferases in thyroid cancer mainly focused on N-acetylgalactosamine-specific α2,6-sialyltransferase 2 (ST6GalNAc2) [36,37]. Miao and Zhao evaluated ST6GalNAc2 protein and mRNA expression in two human FTC cell lines (FTC-238 and FTC-133) and FTC specimens. The expression of ST6GalNAc2 mRNA was higher in FTC-238 invasive cells than in FTC-133 non-invasive cells. The study showed that ST6GalNAc2 activated the invasion in FTC cells by regulating the activity of the phosphoinositide 3-kinase (PI3K)/Akt signaling pathway. An immunohistochemical analysis in FTC specimens of 101 cases revealed a higher expression level of ST6GalNAc2 compared to the normal thyroid tissues. In addition, the study found that elevated expression of ST6GalNAc2 was associated with histological grade, clinical stage, and lymph node metastasis of FTC [36].

Other sialyltransferases that were evaluated in thyroid cancer cell lines are members of the α-2,8-sialyltransferase (ST8SIA) family and α-2,6-sialyltransferase 2 (ST6GAL2). A study by Ma et al. found that the expression of ST8SIA4 was downregulated in highly invasive FTC-238 than that in minimally invasive FTC-133 cells. In contrast, the ST8SIA6 was significantly increased in highly invasive FTC-238 cells compared with FTC-133. Functional analyses of the same study confirmed ST8SIA4 downregulation contributing to the aggressive properties of FTC [38]. In the study of Xu et al., it was demonstrated that ST6GAL2 was aberrantly overexpressed in FTC, which promoted tumorigenesis of FTC in vitro and in vivo [37]. In spite of this, the involvement of ST6GAL1 in the development of thyroid cancer was poorly studied. Data from the Human Protein Atlas portal showed an increase in ST6GAL1 expression in thyroid cancer. However, although 116 cases showed high ST6GAL1 expression in thyroid cancer, 385 cases had low ST6GAL1 expression. Thus, the authors pointed out that ST6GAL1 is not prognostic in thyroid cancer. Although different types of thyroid cancer were included in this project, an analysis of ST6GAL1 expression between different types of thyroid cancers was not conducted.

The strength of our current study is that we analyzed the ST6GAL1 protein expression in different types of well-differentiated thyroid cancers. We decided to conduct this study since our previous GWA studies showed that certain variants of the *ST6GAL1* gene were associated with plasma Tg levels [19,20]. Tg is the most abundant protein in the thyroid gland that releases thyroid hormones by proteolysis [39] and has increased plasma levels in thyroid pathology [40]. ST6GAL1 could affect Tg levels through several molecular pathways. First, this relationship may be mediated with the Wnt/b-catenin signaling pathway, which is activated by ST6GAL1 via the PI3K/Akt/GSK-3b signaling cascade [23]. With decreased ST6GAL1, there is a consequent downregulation of the PI3K/Akt/GSK-3b pathway, lowering Wnt/b-catenin pathway activity. This pathway is known to induce the expression of thyroid transcription factor 1 (TTF-1), which plays a role in Tg gene transcription [41,42]. Hence, reduced pathway activity may lead to lower TTF-1 levels and, subsequently, Tg levels. Second, ST6GAL1 can influence Tg levels through its effect on the thyroid-stimulating hormone (TSH) receptor by adding sialic acid [43]. This modification enhances intracellular cAMP levels [44], promoting TSH receptor activation, which is associated with increased Tg gene expression. Therefore, a decline in ST6GAL1 expression might result in less TSH receptor sialylation and activation, culminating in decreased Tg gene transcription. A third hypothesis concerns the autoregulatory nature of Tg itself, which can modulate its own expression [45]. A study by Sue et al. indicated that Tg with inadequate iodination or sialylation is more effective in activating Tg-mediated signaling pathways [46], including its interaction with the asialoglycoprotein (ASGP) receptor [47]. Therefore, reduced ST6GAL1 expression may lead to lower Tg sialylation, increasing the presence of poorly sialylated Tg, which can enhance Tg-mediated signaling that suppresses Tg gene expression. However, the ASGPR’s involvement in Tg-mediated signaling is not fully understood, necessitating further research to elucidate the signaling events post Tg binding to ASGPR [47,48].

An increase in *ST6GAL1* mRNA levels was also observed in other thyroid pathologies such as Graves’ disease (GD) [49]. The study by Kiljański et al. demonstrated that sialyltransferase mRNA levels and enzyme activity are elevated in the thyroid tissue of patients with GD. Specifically, they reported a significant increase in mRNA for both sialyltransferase-1 (ST6Gal I) and sialyltransferase-4A (ST3Gal I) in GD compared to control groups. A positive correlation was found between an increased sialyltransferase-1 mRNA level and the thyroid-stimulating hormone (TSH)-receptor antibody titer. These findings suggest an upregulation of sialyltransferases in GD, potentially linked to TSH receptor activation, implying a potential role for altered sialylation in the pathogenesis of Graves’ disease.

The scope of future studies should be the elucidation of ST6GAL1—Tg interaction in thyroid cancers and other thyroid diseases. It could be carried out in several ways: (1) through the analysis of ST6GAL1 and Tg co-expression in various types of thyroid cancers and other thyroid pathologies, (2) with the measurement of ST6GAL1 plasma levels in patients with thyroid cancers and other thyroid pathologies and correlation with plasma Tg levels, and (3) with the analysis of the distribution of different ST6GAL1 genotypes associated with plasma Tg levels between patients with different thyroid pathologies and healthy controls. These results should be further validated in larger cohorts and the potential of ST6GAL1 as a biomarker for differential diagnoses of thyroid cancers should be further confirmed.

## 4. Materials and Methods

### 4.1. Tissue Samples of Patients

Thyroid tissue paraffin blocks were collected from 89 patients who underwent thyroidectomy at the University Hospital of Split between April 2019 and December 2022. We examined papillary carcinomas, 20 with classical papillary morphology and 17 follicular variant papillary carcinomas, 18 microcarcinomas, and 20 follicular carcinomas. Normal thyroid tissue samples were obtained from thyroid tissue paraffin blocks of 14 patients who had non-oncological thyroid surgery and had no autoimmune thyroid disorders. All tissues were reviewed by an expert pathologist and were histologically confirmed as carcinomas based on histopathological evaluation. Tissues were processed with the permission of two Ethics Committees, University of Split, School of Medicine (Classification no. 003-08/19-03/0003; Registry no. 2181-198-03-04-19-0022) and University Hospital Split (Classification no. 500-03/19-01/26; Registry no. 2181-147-01/06/M.S.-19-02), in accordance with the Code of Ethics and Helsinki Declaration. Informed consent was obtained from all subjects involved in the study.

### 4.2. Immunofluorescent Staining and Microphotograph Quantification

Formalin-fixed and paraffin-embedded tissue sections were deparaffinized in xylol and rehydrated in ethanol and distilled water. The samples were run through the process of antigen retrieval in a citrate buffer. Nonspecific binding was blocked with Protein Block (Abcam, Cambridge, UK). Tissue sections were then incubated with a primary antibody to ST6GAL1 (1:100, Cat# SAB4502780, RRID: AB_10744544, Sigma-Aldrich, USA, Darmstadt, Germany) overnight at 4 °C. Staining was visualized using incubation with a secondary antibody labelled with green (donkey anti-mouse labelled with AF488, 1:300, Cat# 711-545-152, RRID: AB_2313584, Abcam, Cambridge, UK) fluorochrome. Finally, samples were counterstained with 4′,6-diamidino-2-phenylindole (DAPI).

Photomicrographs were shot with a Nikon DS-Ri1 camera (Nikon Corporation, Tokyo, Japan), mounted on an Olympus BX61 fluorescence microscope (Olympus, Tokyo, Japan). Camera settings were set using image acquisition NIS-Elements F 4.60 software (Nikon Corporation, Tokyo, Japan) at a 1360 × 1024 resolution and exposition of 1/333.3 s (for green fluorescence channel) with a noise reduction filter. Ten microphotographs of thyroid follicles were shot per slide in green and blue fluorescent channels, under the magnification of 20×. Fluorescence-intensity histograms were acquired for the green fluorescence channel in ImageJ 1.53e software (NIH, Bethesda, MD, USA). The region of the positive signal was determined by using the slides stained with secondary antibodies only, thus quantifying the autofluorescence and fluorescence due to the unspecific binding of secondary antibodies. The region of the positive signal was defined as the one that excluded intensities of the signal that were both higher and lower than a signal generated with coupling of primary and secondary antibodies. Expression of ST6GAL1 was quantified as the area under the curve (AUC) of fluorescence-intensity histograms since this measure incorporates both areas under the positive signal and magnitude of signal fluorescence intensity.

### 4.3. ST6GAL1 mRNA Level Analysis

*ST6GAL1* mRNA expression data for thyroid pathologies were obtained from the TCGA cohort (https://www.cancer.gov/tcga, URL (accessed on 30 June 2023), and the *ST6GAL1* mRNA expression data for healthy individuals were from the Genotype-Tissue Expression (GTEx) project [50]. We used the normalized RSEM (RNA-Seq by Expectation Maximization) [51] data on the ST6GAL1 expression.

The data for both cohorts were retrieved from the UCSC Xena database (https://xenabrowser.net/, URL (accessed on 30 June 2023) and processed using the R package “UCSCXenaTools” [52].We also retrieved the phenotypic data for both TCGA and GTEx cohorts, as well as clinical and survival data for the TCGA cohort.

### 4.4. Statistical Analysis

ST6GAL1 protein expression data (i.e., AUC) were normalized to the average of the control group. Welch-corrected one-way ANOVA followed by Dunnett’s T3 multiple comparisons test or *t*-test with Welch correction were used to test the differences in ST6GAL1 protein expression between the groups. When data followed log-normal distribution, logarithmic transformation was applied. Normalized RSEM data were compared between the groups using either a *t*-test or ANOVA. The survival analysis was performed in R using the Kaplan–Meier (KM) method and the log-rank test implemented in the R packages ‘survival’ and ‘survminer’ [53]. A *p*-value less than 0.05 was considered significant. The analysis was performed in R Statistical 4.2.1. Software [54] and GraphPad Prism 9.0 Software (Graph Pad, La Jolla, CA, USA).

## 5. Conclusions

To the best of our knowledge, this is the first study that reveals differential expression of ST6GAL1 in well-differentiated thyroid cancers. We showed that expression of ST6GAL1 was increased in all examined thyroid tumors compared to normal thyroid tissue, with the highest expression in FVPTC. TCGA data also showed elevated *ST6GAL1* mRNA expression in FVPTC and a high association with lymph node metastasis status, clinical stage, and shorter overall survival in thyroid cancers. The scope of future studies should be clarification of molecular pathways through which ST6GAL1 is involved in thyroid cancer.

## Figures and Tables

**Table 1:** ST6GAL1 protein expression and clinicopathological characteristics in patients with thyroid cancers.

		ST6GAL1 Expression		
Variable	N	Median (Q1–Q3)	Min–Max	*p*-Value
**Histological type**				<**0.0001** ^a^
FTC	20	4.94 (3.47–6.45)	1.76–7.73	<**0.0001** ^b^
FVPTC	17	5.47 (3.78–9.44)	1.83–18.33	**0.0002** ^b^
PTC	20	6.34 (2.53–7.83)	0.49–12.40	<**0.0001** ^b^
Micro	18	4.84 (3.26–6.85)	2.64–7.93	<**0.0001** ^b^
Control	14	1.02 (0.56–1.23)	0.10–2.85	
**Clinical stage**				0.299 ^a^
I	51	5 (3–8)	0–18	-
II	8	5 (3–5.75)	3–6	-
III	9	6 (3–7)	2–8	-
IVa	3	5 (2–/)	2–6	-
Missing	4			
**Lymph node status**				0.443 ^a^
Positive	9	5 (3–7)	1–18	-
Negative	63	6 (3.50–9)	0–12	-
Missing	3			-
**Invasion of blood or lymph vessels**				0.396 ^a^
Positive	6	7 (5–10.25)	2–17	-
Negative	48	5 (4–7)	0–18	-
Missing	21			-

ST6GAL1, ST6 beta-galactoside alpha-2,6-sialyltransferase 1; N, number of cases; FTC, follicular thyroid cancer; FVPTC, follicular variant of papillary thyroid cancer; Micro, microcarcinomas; PTC, papillary thyroid cancer; ^a^ Welch’s ANOVA test, ^b^ Unpaired *t*-test with Welch’s correction for the comparison with the control group.

**Table 2:** Clinicopathological characteristics of patients with thyroid cancers in the TCGA cohort.

ST6GAL1 Expression					
Variable	N	Median (Q1–Q3)	Min–Max	*p*-Value	Post Hoc *p*-Value *
**Sample type**				0.008 ^a^	
Primary and metastatic tumors	468	11.25 (10.28–12.33)	7.80–16.67		-
Normal tissue (GTEx)	279	11.12 (10.72–11.51)	0–14.14		-
**Histological type**				**5.50 × 10^−6 a^**	
PTC	366	10.30 (9.48–10.41)	7.20–15.93		-
FVPTC	102	11.52 (10.23–12.61)	7.12–15.81		-
**Clinical stage**				**0.036 ^b^**	
I	273	10.62 (9.64–11.66)	7.42–15.93		I–II: 0.685
					I–III: 0.610
					I–IVa: 0.099
II	51	10.84 (9.70–12.24)	7.27–14.45		II–III: 0.267
					II–IVa: **0.042**
III	92	10.41 (9.62–11.28)	7.20–15.14		III–IVa: 0.592
IVa	42	9.87 (8.95–11.24)	7.68–13.67		
**Lymph node metastasis status**				**1.57 × 10^−4 a^**	
Positive	198	10.26 (9.38–11.25)	7.51–13.67		-
Negative	160	10.82 (9.78–12.07)	7.20–15.81		-
Missing	110	-	-		-

ST6GAL, ST6 beta-galactoside alpha-2,6-sialyltransferase 1; N, number of cases; Min, minimum value; Max, maximum value; Q1, lower quartile; Q3, upper quartile; GTEx, The Genotype-Tissue Expression project; PTC, papillary thyroid cancer; FVPTC, follicular variant of papillary thyroid cancer. ^a^ *t*-test. ^b^ ANOVA test. * *p*-Value of Tukey’s post hoc test.

## Data Availability

The data presented in this study are available on request from the corresponding author.
